# Miscibility of Phosphatidylcholines in Bilayers: Effect of Acyl Chain Unsaturation

**DOI:** 10.3390/membranes13040411

**Published:** 2023-04-05

**Authors:** Agata Żak, Natan Rajtar, Waldemar Kulig, Mariusz Kepczynski

**Affiliations:** 1 Faculty of Chemistry, Jagiellonian University, Gronostajowa 2, 30-387 Kraków, Poland; 2 Department of Physics, University of Helsinki, P.O. Box 64, FI-00014 Helsinki, Finland

**Keywords:** lipid membranes, phase separation, MD simulations, differential scanning calorimetry, Langmuir monolayer measurements

## Abstract

The miscibility of phospholipids in a hydrated bilayer is an issue of fundamental importance for understanding the organization of biological membranes. Despite research on lipid miscibility, its molecular basis remains poorly understood. In this study, all-atom MD simulations complemented by Langmuir monolayer and DSC experiments have been performed to investigate the molecular organization and properties of lipid bilayers composed of phosphatidylcholines with saturated (palmitoyl, DPPC) and unsaturated (oleoyl, DOPC) acyl chains. The experimental results showed that the DOPC/DPPC bilayers are systems exhibiting a very limited miscibility (strongly positive values of excess free energy of mixing) at temperatures below the DPPC phase transition. The excess free energy of mixing is divided into an entropic component, related to the ordering of the acyl chains, and an enthalpic component, resulting from the mainly electrostatic interactions between the headgroups of lipids. MD simulations showed that the electrostatic interactions for lipid like-pairs are much stronger than that for mixed pairs and temperature has only a slight influence on these interactions. On the contrary, the entropic component increases strongly with increasing temperature, due to the freeing of rotation of acyl chains. Therefore, the miscibility of phospholipids with different saturations of acyl chains is an entropy-driven process.

## 1. Introduction

Recent models of biological membranes assume that lipids are not homogeneously distributed within membranes, but form domains or clusters [1,2]. Phosphatidylcholines (PCs) are the main structural lipids in animals and fungi [3]. They constitute 30–60 mol% of all phospholipids in all types of animal cell membranes. Therefore, the study of the phase behavior of PCs, especially their miscibility, is important in understanding the function of biomembranes. In this context, binary mixtures of phospholipids in excess water can be considered the simplest model membranes but, despite their simplicity, their phase behavior can be quite complex.

The miscibility of various PCs in membranes has been extensively studied using both experimental methods and molecular dynamics (MD) simulations. It is generally accepted that membrane composition, temperature, length, and the degree of unsaturation of the acyl chains are the most important factors affecting lipid miscibility. The experimental studies mainly focused on the determination of phase diagrams describing the boundaries between the different states of mixed PC bilayers. A number of methods have been used to construct the phase diagrams, including magnetic resonance and fluorescence spectroscopy, X-ray scattering, and differential scanning calorimetry (DSC) [4]. For example, X-ray scattering was used to determine phase transitions in mixed dioleoylphosphatidylcholine (DOPC)/dipalmitoylphosphatidylcholine (DPPC) membranes [5]. The phase equilibria of DOPC/DPPC-*d*_62_ mixtures in excess water have also been studied using ^2^H NMR techniques [6]. The results of these studies show that above the chain melting temperature of DPPC, fully hydrated mixtures of DOPC and DPPC are in the liquid crystalline phase at all compositions, which is characterized by rapid lateral diffusion of the molecular components, rapid axial diffusion of the molecules about the bilayer normal, and rapid lipid chain isomerization [7]. As the temperature is lowered, samples rich in DPPC enter a two-phase, gel–liquid crystalline coexistence region. The molecular motions are much slower in the gel phases.

Molecular dynamics simulations can provide insight into the organization of lipid membranes at the atomic scale. Several MD studies of binary systems consisting of saturated and unsaturated lipids can be found in the literature [8,9,10,11]. However, most of these present short simulations performed using coarse-grain (CG) or united-atom (UA) force fields (FF) at temperatures above the transition temperature (*T*_m_) values of both lipid components. Using UA-FF, Pyrkova et al. studied membranes containing different proportions of DOPC and DPPC in the liquid phase [8]. They observed non-ideal mixing at the nanoscale. In turn, short-term MD simulations of the DOPC/DPPC membrane using the same force field have shown that DPPC and DOPC mix with little or no excess free energy of mixing at 298 K, which is below the *T*_m_ of DPPC [9]. The near-ideal behavior in such a system is unexpected, given that mixtures of these two lipids are known to separate into fluid (DOPC-enriched) and gel (DPPC-enriched) phases at the simulation temperature, as shown experimentally [6]. Baoukina et al. used MD simulations with the MARTINI CG FF to study membranes formed by POPG, DOPC, and DPPC [10]. At temperatures below the *T*_m_ of DPPC, the system showed the coexistence of DPPC-rich gel domains in a liquid crystalline membrane rich in DOPC and POPG. Using the same FF, Rosetti and Pastorino studied the equimolar binary mixtures of DPPC with diunsaturated phospholipids of different chain lengths in the fluid phase [11]. These membranes showed non-ideal behavior characterized by one mixed phase with compositional fluctuations at the nanometer scale. In summary, there are only a few simulation studies on binary systems with saturated and unsaturated lipids. In addition, the simulations were mostly carried out at temperatures above the *T*_m_ values of both lipid components, and the results obtained show contradictions.

Based on the analysis of the literature, the intermolecular interactions in the mixed PC membranes, and thus their molecular organization, are not fully understood. In this study, we focus on the molecular organization of mixed PC membranes. We are particularly interested in the influence of the saturation of hydrocarbon chains on the miscibility and intermolecular interactions in these membranes. For this purpose, we performed all-atom MD simulations of several systems containing lipids with saturated and unsaturated acyl chains. Langmuir monolayer and DSC experiments were then used to complement the MD simulation results. Our observations showed a complex behavior of both mono- and bilayers, which was determined by the type of hydrocarbon chains (unsaturated or saturated) and thus the effective shape of the lipid molecule. Our research elucidates the thermodynamic basis of domain formation in lipid membranes containing saturated lipids and thus may contribute to a better understanding of the lateral heterogeneity in the lipid composition of cell plasma membranes.

## 2. Material and Methods

**Materials**. Both 1,2-dipalmitoyl-*sn*-glycero-3-phosphocholine (DPPC) and 1,2-dioleoyl-*sn*-glycero-3-phosphocholine (DOPC) were purchased from Avanti Polar Lipids Inc. (Alabaster, AL, USA) with ≥99% purity. Chloroform (HPLC grade, ≥99.9%) and phosphate-buffered saline (PBS, tablets) were obtained from Merck Life Science Sp.z.o.o. (Poznań, Poland). The ultrapure Milli-Q water used in the experiments had a surface tension of 72.6 mN/m (at 20 °C) and a resistivity of 18 MΩ cm.

**Langmuir Monolayer Experiments**. The experiments were carried out using a KSV 2000 Langmuir trough (KSV Instruments Ltd., Helsinki, Finland), as previously described [12]. Briefly, lipids were dissolved in chloroform to form stock solutions. They were stored at −20 °C and mixed in the required proportions in glass vials just before the experiments. The lipid mixtures were deposited on a subphase of ultrapure water using a Hamilton analytical syringe. After spreading, the monolayers were left for 5 min and then compression was initiated at a barrier speed of 10 mm/min. The subphase temperature was maintained at 22 °C using a Julabo circulating water bath. All experiments were repeated at least twice to ensure the consistency of the results. The isotherms were analyzed as previously described [12] and two values were calculated: (i) the compression modulus (C_S_^−1^) which provides information about the physical state of the films, their elasticity, and the ordering of molecules during compression, and (ii) the excess free energy of mixing (Δ*G*^Exc^) which describes the interactions in the mixed films.

**Differential Scanning Calorimetry (DSC)**. DSC experiments were performed using a NanoDSC TA Instruments calorimeter (New Castle, DE, USA) with 0.3 mL capillary cells. Lipid suspensions in PBS (2 mg/mL total lipid concentration and varying DOPC mole fractions, *X*_DOPC_) were measured from 5 to 50 °C at a scan rate of 0.5 °C/min. Thermograms for the buffer were obtained under identical conditions and subtracted from the excess heat capacity curves. *T*_m_ was defined as the temperature at the peak maximum.

**Figure 1:**
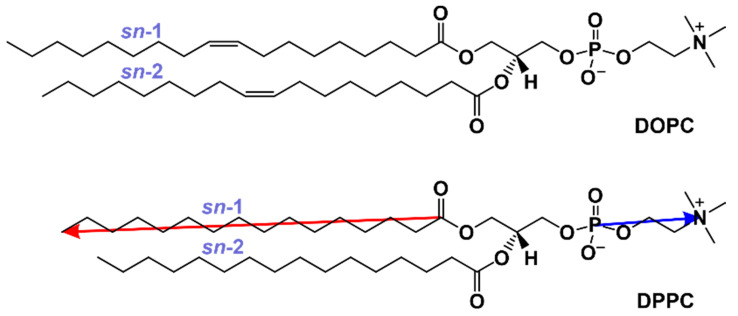
Chemical structures of lipids used in this study. The lipid chain vector and headgroup vectors, used in the orientation analyses, are shown as red and blue arrows, respectively.

**MD Simulations**. All-atom MD simulations of lipid bilayers consisting of DOPC, DPPC, and DPPC/DOPC mixtures with *X*_DOPC_ equal to 0.1, 0.3, 0.5, 0.7, and 0.9 were performed. The chemical structures of the lipids are shown in Figure 1, while a detailed description of the components of all simulated systems is given in Table 1. All systems were simulated at 22 °C and 50 °C (i.e., below and above the DPPC main phase transition temperature *T*_m_ = 41 °C). Initial structures of lipid membranes were constructed by placing lipids in random orientations on an 8 × 8 grid, resulting in lipid bilayers containing 128 lipids (two leaflets of 64 lipids in each). After solvation, the systems were first energy minimized using the steepest descent algorithm, followed by the MD simulations for 1 µs in the isobaric–isothermic (NpT) ensemble. The representative time evolution of the thermodynamic and structural parameters from MD simulations for the system DPPC/DOPC with *X*_DOPC_ = 0.9 at 295 K is shown in Figure S2. The first 500 ns of the simulation was treated as the equilibration period; thus, only the last 500 ns of each trajectory was analyzed unless otherwise stated. Each simulation was repeated three times, starting from different initial configurations of lipids and water molecules.

An all-atom optimized parameters for liquid simulations (OPLS-AA) force field [13,14] with additional parameters developed by us was used to parametrize lipid molecules [15,16,17]. For water, the TIP3P model was employed [18]. All MD simulations were carried out at a constant pressure of 1 bar using the Parrinello–Rahman algorithm [19] with a pressure constant of 1 ps. A semi-isotropic algorithm was applied for pressure. The lipid bilayer and water temperatures were set to 295 K or 323 K and controlled independently by the Nose–Hoover algorithm [20,21] with a temperature constant of 0.4 ps. Periodic boundary conditions in all three directions were used. The LINCS algorithm [22] was used to preserve covalent bonds with the simulation time step of 2 fs. For water molecules, the SETTLE method [23] was used. Long-range electrostatic interactions beyond the cutoff of 1 nm were treated with the particle mesh Ewald algorithm [24,25]. All MD simulations were performed using the GROMACS 2020.5 software package [26]. Visualizations of the simulated systems were performed using Visual Molecular Dynamics (VMD) software [27].

## 3. Results and Discussion

The miscibility of phospholipids in a hydrated bilayer is an issue of fundamental importance to membrane biophysics [28]. This research aimed to investigate the impact of the unsaturation of lipid acyl chains on the molecular organization of mixed lipid membranes composed of phosphatidylcholines (PCs), and to understand the driving forces causing the immiscibility of PCs. It has previously been shown experimentally that DOPC and DPPC exhibit limited miscibility in a mixed bilayer [5]. Therefore, we used this lipid pair to further explore the reason for differences in immiscibility between PC molecules. Both lipids share the same headgroup but differ in the length and saturation of the acyl chains. This enabled us to isolate the effect of the hydrophobic part of the lipids on their behavior in the mixed bilayers.

**DSC Measurements**. DSC measurements were used to determine the phase diagram for the mixed DPPC/DOPC bilayers. DSC thermograms for systems with various *X*_DOPC_ are depicted in Figure S1. The thermogram for the pure DPPC membrane displays two endothermic peaks at *T*_p_ = 35 °C and *T*_m_ = 41.2 °C. The first peak corresponds to the pre-transition from the gel phase (*L_β_*_′_, also called the solid-ordered phase) to the ripple phase (*P_β_*_′_). In the *L_β_*_′_ state, the lipids are arranged on a two-dimensional triangular lattice in the plane of the membrane [1]. DPPC chains are predominantly ordered into the all-*trans* configurations and tilted with respect to the membrane normal. For fully hydrated DPPC bilayers at 19–25 °C, the tilt angle is about 30° [29]. The *P_β_*_′_ phase is characterized by periodic one-dimensional ripples on the membrane surface. The ripples are probably formed by periodic arrangements of the linear gel (*L_β_*_′_) and fluid (*L_α_*) lipid domains [30,31]. The second peak corresponds to the main transition (membrane melting) from the *P_β_*_′_ phase to the liquid phase (*L_α_*, also known as the liquid-disordered or liquid-crystalline phase). The main transition peak is narrow, which indicates that the melting is strongly cooperative. DPPC molecules melt simultaneously in large clusters of *n* lipids (these *n* lipids are considered as the size of the cooperative unit) [1]. The larger the size of the cooperative unit, the narrower the transition peak. In the *L*_α_ phase, the acyl chains are mostly disordered with many *trans*- and *gauche* configurations along the chain, and the lattice order is lost [1]. In turn, the DOPC membrane has a main transition temperature of −16.5 °C [32]. This means that the DOPC bilayer is in the liquid phase over the whole temperature range studied here.

The admixture of DOPC into the DPPC bilayer resulted in alterations in the gel-to-liquid state transition. The pre-transition peak disappeared, while the main transition peak became broader, and the *T*_m_ value decreased with increasing DOPC content in the membrane. This shows that the various lipid species in mixtures do not melt independently but rather influence each other in the melting process, and that the presence of DOPC affects the DPPC melting process. The addition of DOPC to the DPPC membrane leads to a broadening of the heat capacity profiles and thus a reduction in the DPPC cooperative unit size.

**Figure 2:**
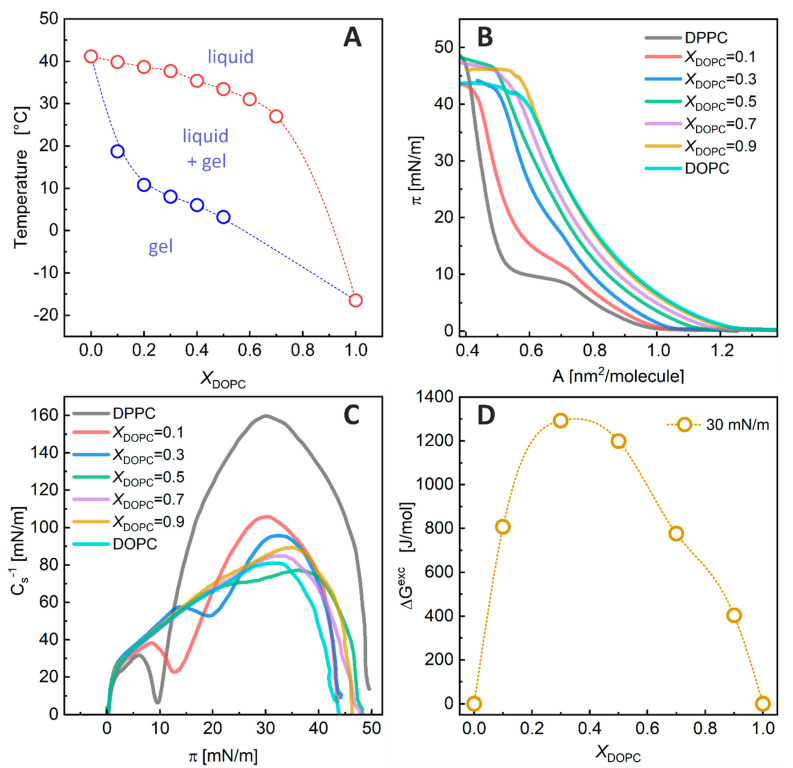
(**A**) Phase diagram of the DPPC/DOPC membrane determined using DSC. The melting temperature of pure DOPC was taken from ref. [32]. (**B**) Surface pressure (π)—area (**A**) isotherms for DPPC/DOPC mixed monolayers measured at 22 °C. Water was used as the subphase. (**C**) Compression modulus versus π for the DPPC/DOPC mixed monolayers at 22 °C. (**D**) Values of excess free energy of mixing (ΔG^Exc^) as a function of the DOPC mole fraction (*X*_DOPC_) at π = 30 mN/m and 22 °C.

A pseudo-binary phase diagram was constructed from the thermograms by plotting the transition temperatures from a single-phase state to the coexistence of two phases as a function of the sample composition. The transition onset and completion temperatures were determined by analyzing the heat capacity curves using the tangent method [33]. Figure 2A shows the phase boundaries among the gel phase, liquid-crystalline phase, and their coexistence region for the DPPC/DOPC membrane. The phase diagram indicates that at 50 °C, the DPPC/DOPC membranes of any composition were in the liquid phase. In contrast, at 22 °C, the systems containing *X*_DOPC_ ≤ 0.1 were in the gel phase and those with *X*_DOPC_ ≥ 0.8 were in the liquid-crystalline state, while the coexistence of both phases was observed within these limits.

**The DPPC/DOPC phase diagram**. Our phase diagram determined for multilamellar vesicles shows that at 22 °C, the DPPC/DOPC membranes with *X*_DOPC_ ≤ 0.1 are in the gel phase, and those containing *X*_DOPC_ ≥ 0.8 are in the liquid phase, while the coexistence of liquid–gel phases is observed within these limits. As expected, for temperatures higher than 41 °C, the mixed DPPC/DOPC bilayers are solely in the liquid state. Due to the nature of DSC measurements, we were only able to determine a part of the phase diagram (for temperatures higher than 5 °C). A more detailed phase diagram for DOPC/DPPC-*d*_62_ mixtures was previously determined using ^2^H NMR experiments [6]. According to this phase diagram, two-phase systems at 22 °C for large multilamellar DOPC/DPPC-*d*_62_ liposomes at pH 7.0 were observed in the range of 0.3 < *X*_DOPC_ ≤ 0.65. Using X-ray scattering, Furuya and Mitsui determined the phase diagram for DPPC/DOPC multilamellar vesicles [5], which indicated that the liquid–gel phase coexistence occurs within 0.1 < *X*_DOPC_ ≤ 0.7. In turn, the fluorescence measurement revealed that this range could change depending on the method of liposome preparation [34]. Therefore, the observed difference in phase diagrams for mixed lipid membranes may be due to the difference in the method of liposome preparation, as well as due to the experimental method used in the measurements. The DSC experiments used in this study clearly showed that at *X*_DOPC_ = 0.7, there are still DPPC domains that melt with increasing temperature, so the DPPC/DOPC membranes with this composition undergo phase separation at lower temperatures.

**Langmuir Monolayer Experiments**. The measured π–A isotherms and the values of the compression modulus (C_S_^−1^) calculated on their basis are shown in Figure 2B,C, respectively. The isotherms for the pure DPPC and DOPC monolayers are consistent with those previously published [12,35]. The shape of the π–A isotherm for the DOPC film and its C_S_^−1^ − π dependency (Figure 2C) indicate that this monolayer is in the liquid (L) phase. For DPPC, an inflection region in the isotherm and a minimum in the C_S_^−1^ vs. π plot indicate that this monolayer changes its physical state during compression. The phase transition corresponds to a change from the liquid-expanded (Le) to the liquid-condensed (Lc) state. The addition of DOPC to the DPPC monolayer shifts the Le-Lc phase transition towards higher surface pressures (π_Le-Lc_ = 9.6, 12.8, 19.6, and ~30 mN/m for *X*_DOPC_ = 0.0, 0.1, 0.3, and 0.5, respectively), indicating that the mixed DOPC/DPPC films are less condensed than the pure DPPC monolayer. For *X*_DOPC_ ≥ 0.7, the inflections resulting from the Le-Lc phase transition vanished in the isotherms—indicating that at 22 °C, the monolayers of these compositions are in the L phase, similar to the pure DOPC film. 

It has been shown that the properties of lipid monolayers at surface pressures in the range of 30–35 mN/m correspond to the properties of bilayers [36]. Therefore, to shed more light on the miscibility of monolayer components, the A values at a surface pressure of 30 mN/m were determined from the isotherms, and the values of ΔG^Exc^ [37] were calculated and plotted versus the DOPC mole fraction (Figure 2D) to compare the magnitude of interactions between components in the DPPC/DOPC monolayers. The excess mixing free energy values were positive for all the DPPC/DOPC monolayers, showing that the intermolecular interactions in the mixed films were unfavorable compared to those in the one-component films. The strongest repulsion between lipid molecules was observed for an *X*_DOPC_ in the range of 0.3–0.5. The maximum of ΔG^Exc^ was about 1.3 kJ/mol. 

**The excess free energy of mixing**. Our experiments showed a significant influence of the saturation of the lipid chains on the mutual miscibility between PCs. A comparison of one-component DPPC and DOPC monolayers showed that DPPC molecules are more densely packed in the monolayer, and thus more ordered than DOPC molecules in their films, due to the bending of the hydrocarbon chains in the latter lipid. Miscibility between DPPC and DOPC lipids is thermodynamically unfavorable in all mixed membrane compositions, as the values of the excess free energy of mixing are positive and reach a maximum of 1.3 kJ/mol at an *X*_DOPC_ of about 0.3–0.5.

We previously investigated miscibility in mixed phosphatidylcholine (PC)–phosphatidic acid (PA) bilayers in which the lipids differed in their degree of unsaturation [12,35]. In the case of the DPPC–DPPA system, the maximal value of the excess free energy of mixing was about −0.5 kJ/mol, indicating that the interactions between the components are more attractive than for the pure DPPC and DPPA monolayers. BAM images for selected DPPC–DPPA film compositions, showing monolayer morphology, demonstrated that the film components are miscible over the entire range of monolayer compositions. For DOPC–DOPA mixtures, the ΔG^Exc^ values were only slightly positive (<0.08 kJ/mol), and the monolayers were completely homogeneous for all film compositions. This indicates an ideal mixing between DOPC and DOPA. In contrast, the mixture of DOPC with DPPA showed large positive deviations from the ideal, and the maximal ΔG^Exc^ value was ca. 1.7 kJ/mol, indicating that the mixing of DOPC and DPPA molecules is thermodynamically unfavorable. Phase separation resulting from the immiscibility of the monolayer components was clearly visible in the BAM images, where, regardless of the monolayer composition, bright condensed DPPA domains dispersed in the fluid DOPC matrix were visible. More complicated behavior was observed for the DPPC/DOPA mixed monolayers [12]. The positive ΔG^Exc^ values for films containing higher amounts of DOPA (about 0.4 kJ/mol) indicated that lipid miscibility was not thermodynamically favorable. However, the BAM images showed no signs of phase separation. This means that the repulsive interactions between the mixed pairs were rather weak to cause the immiscibility of the DPPC/DOPA membrane. At a low DOPA content (*X*_DOPA_ ≤ 0.3), the mixing of DPPC and DOPA lipids in monolayers was thermodynamically favorable due to the strong attractive electrostatic interactions between their headgroups, resulting in monolayer condensation. The maximal ΔG^Exc^ value for the DPPC/DOPC system is comparable to that for the DOPC/DPPA system. This indicates that the structure of the acyl chains of individual lipids has a much greater impact on their miscibility compared to possible interactions within the headgroups. In conclusion, phase separation is observed when ΔG^Exc^ exceeds a certain limit. Based on the aforementioned values, we suppose that this limit is in the range of 0.8–1.2 kJ/mol. For positive ΔG^Exc^ values which remain below this limit, small lipid aggregates may form, but these are too small to be observed by BAM methods. However, this hypothesis requires further experimental studies.

**Figure 3:**
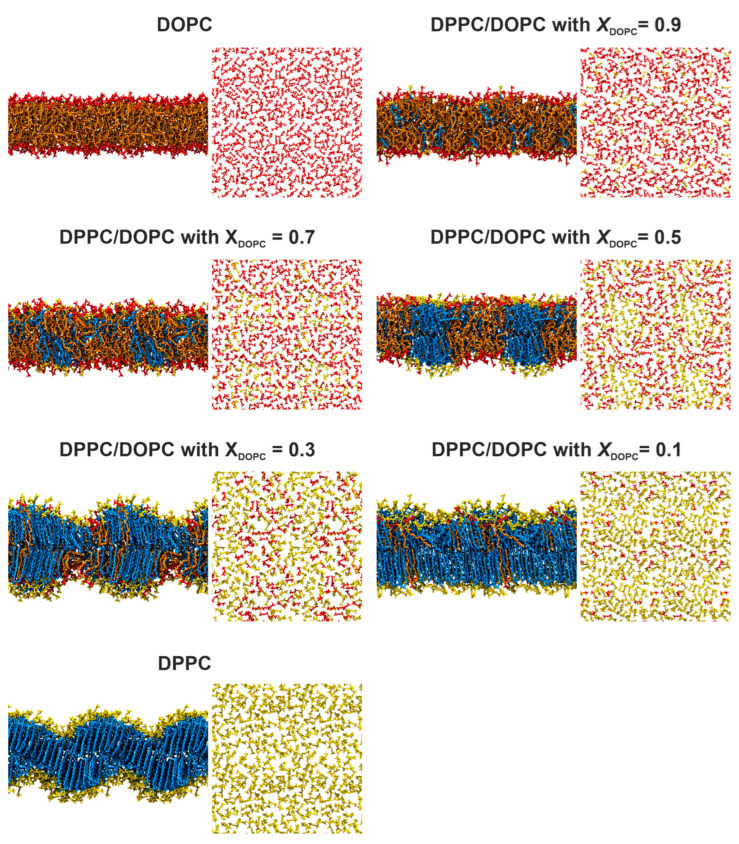
Representative snapshots from MD simulations showing the organization of lipid membranes with different lipid compositions at 295 K. Lipids are shown in the licorice representation. DOPC is shown in orange (hydrocarbon chains) and red (headgroups), while DPPC is shown in blue (hydrocarbon chains) and yellow (headgroups). Water molecules are not shown for clarity.

**MD Simulations**. We performed MD simulations of mixed DPPC/DOPC bilayers with different lipid compositions at 22 °C (295 K) and 50 °C (323 K). Figure 3 and Figure S2 present typical snapshots showing the organization of the DPPC/DOPC membranes at the end of the simulation at 295 K and 323 K, respectively. At the higher temperature, all the bilayers exhibit a pronounced disorder of the acyl chains with a large number of *gauche* configurations, which is characteristic of membranes in the liquid phase. The lipid molecules are evenly distributed in the mixed bilayer, which indicates a good miscibility of the lipids at temperatures above the phase transition. On the contrary, at the lower temperature (Figure 3), the DPPC membrane shows a structure that resembles the ripple phase (*P*_β′_). The presence of two regions with different molecular ordering patterns can be observed. In one region, the DPPC molecules are stretched and tightly packed in a manner characteristic of the gel phase. The two leaflets are well separated, resulting in increased membrane thickness. In the other region, the acyl chains are disordered, and the leaflets are partially interdigitated, which reduces the thickness of the bilayer.

For the mixed DPPC/DOPC bilayers at 295 K, containing 50 mol% or more DPPC, two regions with very distinctive molecular ordering patterns were observed. The first region contains tightly packed lipid molecules (mainly DPPC) with stretched chains arranged similarly to the pure DPPC bilayer described above. The second region contains loosely packed lipid molecules (mostly DOPC) with partially interdigitated hydrocarbon chains. This molecular arrangement changes for the bilayers with a lower DPPC content (*X*_DOPC_ = 0.7 and 0.9). In these cases, there is no clear phase separation between DPPC and DOPC lipids, although DPPC molecules seem to adhere to each other to form small aggregates. The lipid bilayers are much more disordered and there is no clear separation between lipid leaflets.

**Area per lipid**. In order to gain more insight into the lipid organization in the DPPC/DOPC bilayers, the average area per lipid (APL), the average area per DPPC (APP), and the average area per DOPC (APO) were calculated (Table 2). APL was computed by dividing the area of the simulation box by the number of lipids in one leaflet, while the APP and APO in each membrane were calculated using the Voronoi tessellation method implemented in the APL@VORO software [38]. 

The calculated APL value for the pure DPPC membrane is 0.664 nm^2^ at 323 K. This value is very close to experimental results (ca. 0.629 [39], 0.64 [40], 0.681 [41], and 0.68 [42] nm^2^) determined at the same temperature. Similarly, the calculated APLs for the pure DOPC bilayer are 0.744 nm^2^ and 0.722 nm^2^ for 323 K and 295 K, respectively, and are also in good agreement with the experimental values of 0.721 nm^2^ [41] and 0.722 nm^2^ [43] measured at 298 K and 303 K, respectively. As expected, an increase in DOPC content in the mixed membrane leads to a gradual increase in APL. The average values of APP and APO also increase with the increasing DOPC concentration in the bilayer. At lower temperature, APPs are much smaller compared to APOs for *X*_DOPC_ ≤ 0.5, but for higher unsaturated lipid content both values become similar. This shows that in the membranes with *X*_DOPC_ > 0.5, the palmitoyl chains of DPPC become more disordered, which increases the surface area occupied by this lipid. At a higher temperature, the differences between APL and APP or APO are insignificant.

It should be noted that the average value of area per DPPC (APP) in the pure DPPC bilayer at 323 K is lower than the average value of the area per lipid (APL). The same is true for the APO and APL values for the pure DOPC bilayer. This difference is because the Voronoi tessellation method does not take into account voids between lipids when reporting the area per lipid (APO and APP), while the average APL values include them. At the higher temperature, the differences between APL and APP or APO are greater than those at the lower temperature, confirming that at the higher temperature the lipids are further apart, making the structure much looser.

**Figure 4:**
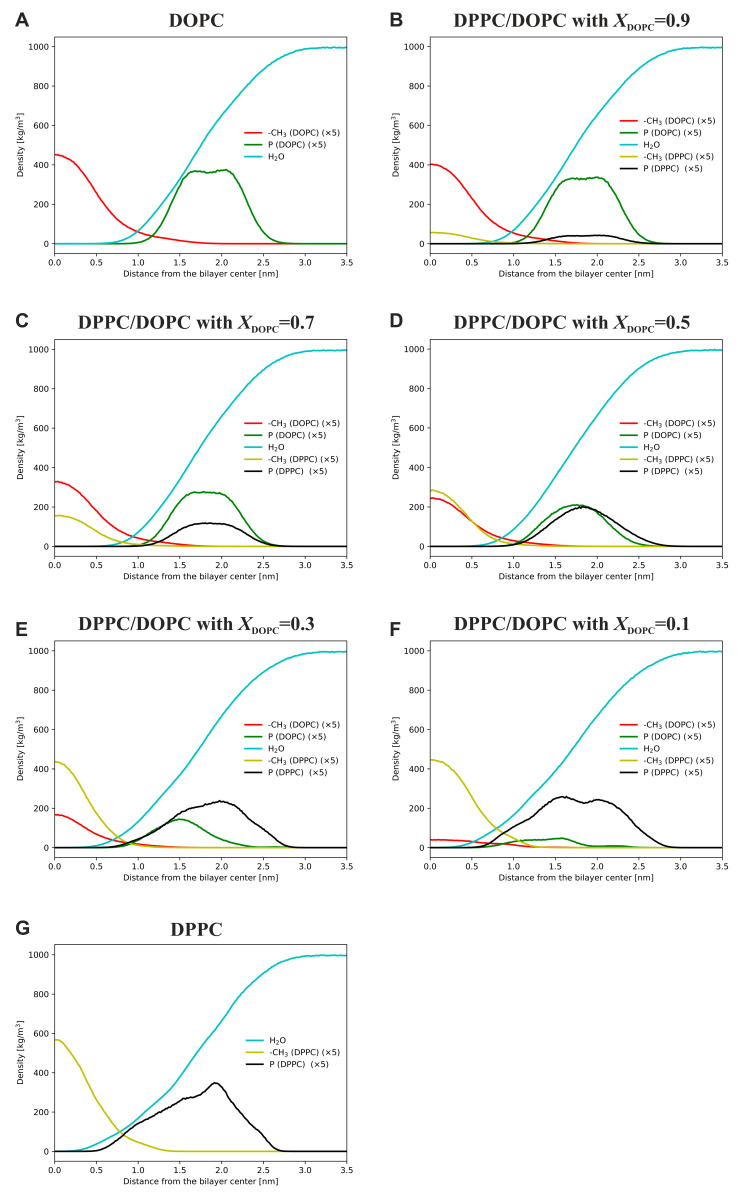
Mass-density profiles of lipids and selected chemical groups in lipid membranes at 295 K for DOPC (**A**), DPPC/DOPC with *X*_DOPC_ = 0.9 (**B**), DPPC/DOPC with *X*_DOPC_ = 0.7 (**C**), DPPC/DOPC with *X*_DOPC_ = 0.5 (**D**), DPPC/DOPC with *X*_DOPC_ = 0.3 (**E**), DPPC/DOPC with *X*_DOPC_ = 0.1 (**F**), and DPPC (**G**). The density profiles of water (cyan curve), phosphorus atoms (green and black curves for DOPC and DPPC, respectively) and the terminal methyl group of hydrocarbon chains (red and yellow curves for DOPC and DPPC, respectively) are shown.

**Membrane thickness**. The mass density profiles for selected lipid groups for the systems simulated at 295 K are depicted in Figure 4. These profiles show the distributions of the various lipid groups along the normal to the bilayer. The distributions of DOPC phosphorus atoms are shifted towards the center of the mixed bilayer compared to the distributions of DPPC phosphorus atoms (Figure 4). This is particularly evident for *X*_DOPC_ < 0.7 and suggests that DOPC lipids are, on average, located deeper in the mixed lipid bilayer compared to DPPC molecules. At 323 K (Figure S3, Supplementary Materials), this effect is much weaker, showing that at the higher temperature the headgroups of both lipids are in similar positions in the membrane. The distributions of DPPC phosphorus atoms at 295 K are much more asymmetric compared to the distributions of the DOPC phosphorus atoms. This trend disappears at 323 K, where the distributions of DPPC phosphorus atoms become fully symmetric. Interestingly, the water-mass-density profiles (Figure 4) show that the depth of the water penetration deep into the lipid bilayer increases with decreasing DOPC content in the mixed membranes. This effect is more pronounced at lower temperatures.

The bilayer thickness (*d*_P_) was calculated as an average distance between the phosphorus atoms in the opposite leaflets for each type of lipid separately (Table S1). The calculated *d*_P_ values for pure DPPC and DOPC membranes are in good agreement with the experimental results. The bilayer thickness of DPPC, DOPC, and an equimolar mixture of these two phospholipids at 50 °C was determined experimentally (using X-ray diffraction measurements) to be 4.02, 3.64, and 3.74 nm, respectively [44]. It follows that the thickness of the bilayer formed by the equimolar mixture is intermediate between the two pure phospholipid bilayers. The thickness of mica-supported DPPC and DOPC bilayers was previously determined using atomic-force microscopy (AFM) [45]. For DOPC, the AFM measurements revealed a membrane thickness of about 4.1 nm at 22 °C. In the case of DPPC, AFM topography images showed a bilayer thickness of 4.8 ± 0.3 nm at 22 °C (gel phase), and this value decreased to 3.3 ± 0.3 nm when the temperature was increased to 52 °C. The authors noted that the membrane thicknesses obtained from the cross-section of AFM images at room temperature were larger than the values determined by other experimental methods. As an explanation, they stated that AFM can overestimate the thickness of the lipid membrane at lower temperatures due to the reduced mobility of both lipids and water molecules associated with the bilayer interface.

At the lower temperature, the membrane thickness with respect to DOPC is almost constant in all simulated bilayers and is significantly smaller than that for DPPC. For DPPC, the *d*_P_ value changed with the composition of the mixed bilayer. It first increases to a value of about 4.3 nm at *X*_DOPC_ = 0.3 and then decreases to a constant value of about 3.9 nm. Such a non-linear relationship is probably related to the fact that in our simulations the pure DPPC bilayer has a ripple phase structure (see Figure 2), where the membrane thickness varies periodically. Therefore, the averaged *d*_P_ value is underestimated. The introduction of DOPC leads to the gradual disappearance of the ripple structure, so the resulting average value reaches a value characteristic of the thickness of the DPPC membrane in the solid phase. At 323 K, the thickness of the mixed membrane is constant for both lipids, with the *d*_P_ value for DPPC tending to be larger compared to DOPC, indicating that the unsaturated lipid molecules are located slightly deeper in the membrane. Despite the good agreement between the experimental and calculated values of the bilayer thickness, it is worth noting that the choice of the force field and the method of calculating bilayer thickness (phosphorus-to-phosphorus distance vs. hydrophobic thickness) affect the calculation outcome.

**Figure 5:**
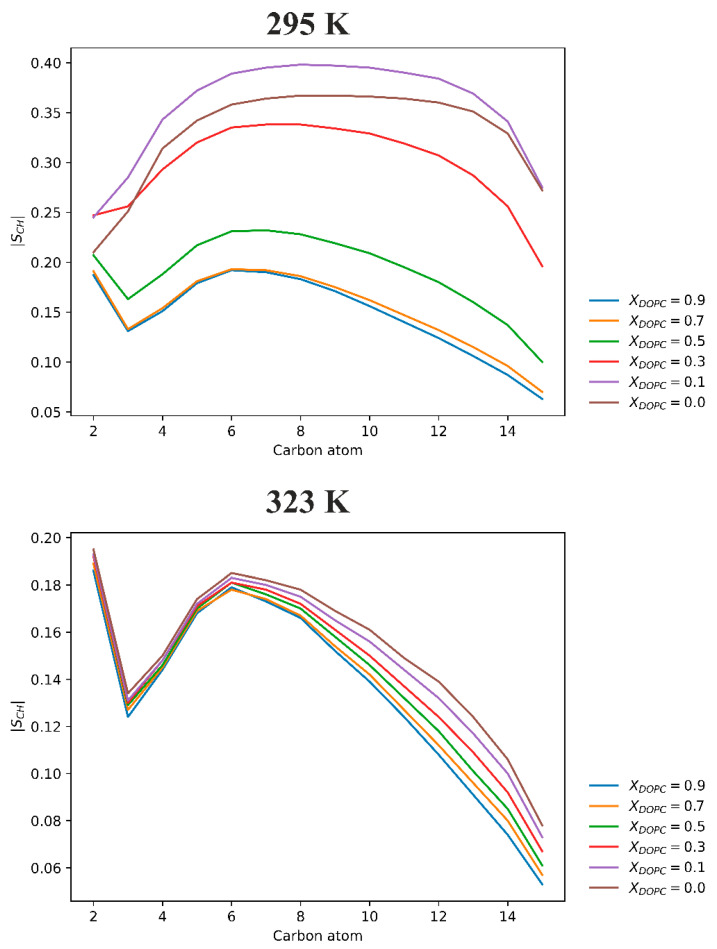
The hydrogen order parameters (S_CH_) profiles of the *sn*-1 chain of the DPPC lipid in the DPPC/DOPC bilayer as a function of the DOPC mole fraction (*X*_DOPC_) at 293 K (**top** panel) and 323 K (**bottom** panel).

**Ordering of lipid acyl chains**. The profiles of the hydrogen order parameters (*S*_CH_) along the *sn*-1 chains of DPPC lipids were then calculated as previously described [35]. Figure 5 shows that, at the higher temperature, the presence of DOPC mainly reduces the ordering of the deeper segments of the DPPC chains (C10–C14 carbons). The effect of the unsaturated lipid on *S*_CH_ in the upper regions of the mixed bilayer (i.e., the most ordered part of the bilayer) is negligible. On the contrary, at 295 K, the addition of DOPC has a significant impact on the ordering of DPPC hydrocarbon chains. The *S*_CH_ value strongly decreases as the mole fraction of DOPC increases. The greatest decrease in the ordering of the mixed DPPC/DOPC membrane is observed when *X*_DOPC_ = 0.3 is exceeded. Interestingly, the addition of 10 mol% DOPC increases the ordering of the DPPC hydrocarbon chains relative to the pure bilayer. This can be explained by the fact that the pure DPPC membrane in our simulations is in the ripple phase (Figure 2), in which some of the lipids show high disorder. The introduction of DOPC causes a partial disappearance of the ripple, and thus an increase in the ordering of the DPPC alkyl chains. 

**Figure 6:**
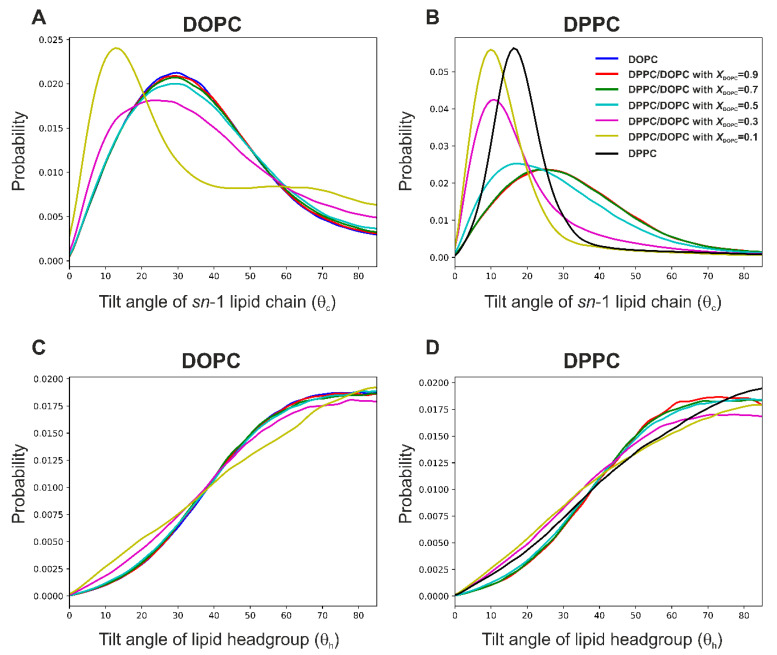
Probability distributions of the tilt angles *θ*_c_ between the *sn*-1 lipid chain vector (shown in Figure 1) and the bilayer normal for DOPC (**A**) and DPPC (**B**), and probability distributions of the angle *θ*_h_ between the headgroup vector and the bilayer normal for DOPC (**C**) and DPPC (**D**) at 295 K. The probabilities were averaged over the time and number of repeats.

**Orientation of lipids in the mixed membrane**. In order to describe the arrangement of the lipid molecules inside the mixed membranes, we calculated the tilt angle (*θ*_c_) between the acyl chains (both *sn*-1 and *sn*-2) and the normal to the membrane, and the tilt angle (*θ*_h_) between the headgroups of DPPC and DOPC lipids and the bilayer normal (see vector definitions in Figure 1). The normalized probability distributions of the angles are shown in Figure 6 and Figure S5 for 295 K and 323 K, respectively.

In all membranes at the lower temperature, the *θ*_c_ distributions for DOPC are wider than the corresponding distributions for DPPC, and the maxima are shifted toward larger angles (Figure 6A,B). This is probably due to the bending of the hydrophobic chain caused by the presence of the double bond. The exception is the membrane with *X*_DOPC_ = 0.1, in which the DOPC molecules adopt an orientation similar to DPPC. In turn, in the lipid bilayers containing more DOPC (*X*_DOPC_ > 0.5), the average tilt angle of DPPC acyl chains becomes similar to that for DOPC. Interestingly, the DOPC acyl chains can adopt a nearly perpendicular orientation with respect to the bilayer normal (non-zero probability for angles above 80°, Figure 6A), while the DPPC acyl chains do not exhibit such behavior. The *θ*_h_ distributions (Figure 6C,D) suggest that the most probable orientation of the headgroups of both DPPC and DOPC is their alignment almost parallel to the membrane surface, but the wide width of the distributions indicates a large degree of freedom in the arrangement of these groups. 

**Figure 7:**
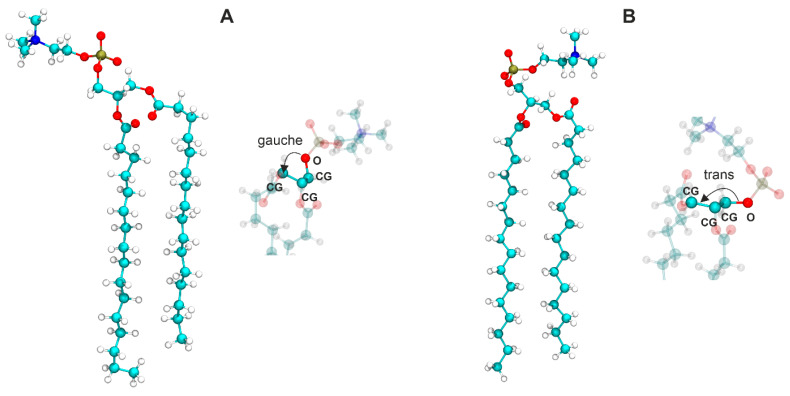
Two DPPC conformations taken from the simulation of the DPPC membrane in the gel phase (295 K). Structure (**A**) corresponds to the situation where the CG-CG-CG-O angle is the *gauche* configuration and structure (**B**) is the situation where the CG-CG-CG-O angle is in the *trans* configuration (CG = glycerol carbon). On the right side of each panel is a schematic representation of the *gauche* or *trans* conformation.

**Figure 8:**
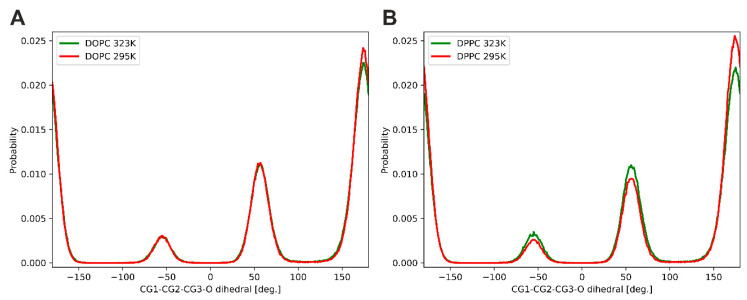
Distributions of *gauche* and *trans* configurations of the glycerol moieties in the equimolar mixture (*X*_DOPC_ = 0.5) of DOPC (**A**) and DPPC (**B**) at 295 K and 323 K.

**Orientation of headgroups in the mixed membrane**. We then focused on the arrangement of the headgroups relative to the entire lipid molecule. Two conformations with different headgroup orientations have been identified in the crystal structure of 1,2-dimyristoyl-*sn*-glycero-3-phosphatidylcholine (DMPC) by X-ray scattering [46]. Conformation A shows the headgroup pointing away from the rest of the molecule, while the headgroup in conformation B is folded back, resulting in a more compact structure. These conformations correspond to different orientations of the glycerol moiety in the lipid molecule. The situations where the CG-CG-CG-O dihedral angles (CG stands for carbon from the glycerol moiety) are in *gauche* and *trans* configurations are described by the structures A and B, respectively. Figure 7 shows the two conformations adopted by the DPPC molecules in the gel state taken from the MD simulations of the DPPC system. The headgroups can be oriented either above or outside the lipid molecule; however, in both cases, they are arranged almost parallel to the membrane surface. The distributions of the *gauche* and *trans* configurations in the equimolar mixture of DPPC and DOPC at 295 K and 323 K are shown in Figure 8. All distributions have three maxima; those at −60° and 60° correspond to the *gauche* configurations of the CG-CG-CG-O dihedral angles (conformation A), while the maximum at about 180° is associated with the *trans* configuration (conformation B). Our results show that both lipids have a lot of freedom to change the arrangement of the headgroups, but the *trans* configuration (with the choline group located above the lipid molecule shielding the hydrophobic part from contact with water) dominates. The contribution of the *trans* configuration decreases with temperature for both lipids, but this effect is stronger for DPPC. 

**Figure 9:**
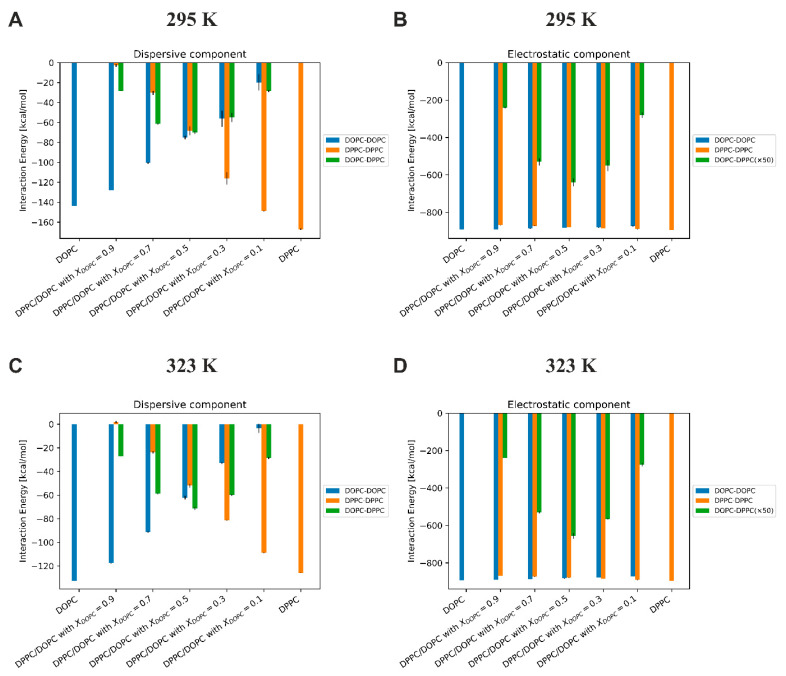
The electrostatic and dispersive components of the interaction energy in the DPPC/DOPC bilayer as a function of the DOPC mole fraction (*X*_DOPC_) at 293 K (panels (**A**,**B**)) and 323 K (panels (**C**,**D**)). All energies were calculated as averages over time and the number of repeats.

**Energy of interactions between lipids**. In order to quantify local interactions between lipids in the mixed DPPC/DOPC bilayers, we calculated the interaction energy between lipid molecules, distinguishing between electrostatic and dispersive components (Figure 9). The dispersion energies, calculated from MD simulations, contain all the terms from the Lennard–Jones interaction potential, which includes both dispersive energy (always attractive) and Pauli repulsion (acting at much shorter interatomic distances). All energies were calculated as averages over time and the number of repeats. 

Several conclusions can be drawn about the interactions between lipids in the mixed DOPC/DPPC membrane from Figure 9: (i) the most important outcome is that temperature has only a slight influence on the value of both dispersive and Coulomb interactions; (ii) the dispersive interactions are much weaker compared to electrostatic interactions (as much as six times as in the case of the pure DOPC membrane); (iii) for the membrane with *X*_DOPC_ = 0.5, the energy of dispersion interactions between all lipid molecules is similar, while the Coulomb interactions for lipid like-pairs are more than 75 times stronger than for mixed-pairs.

**The molecular organization of mixed DPPC/DOPC membrane**. Our simulation results are consistent with experimental findings. The exception is the DPPC pure membrane simulated at 22 °C, which the simulations show is in the ripple phase, which is not in line with the DSC findings. All mixed DPPC/DOPC membranes simulated at temperatures above the DPPC phase transition were in the liquid phase; this means that the acyl chains of both lipids were highly disordered, as indicated by the order parameters (Figure 5). In addition, the distributions of individual lipids in the bilayer were uniform, so the DPPC/DOPC membranes were single-phase systems. 

At the lower temperature, for *X*_DOPC_ = 0.1, a densely packed membrane was observed with DOPC molecules dispersed evenly among DPPC lipids (Figure 3), showing that this mixed membrane is a single-phase system. Importantly, the DOPC molecules adopt the orientation of the surrounding DPPC membrane (Figure 6A), but their headgroups are located deeper compared to those of the saturated lipid. The APO value is much smaller compared to that in the pure DOPC membrane, showing that DOPC molecules adopt a more compact conformation in the mixed membrane. Interestingly, the introduction of a small amount of DOPC results in the disappearance of the ripple structure of the DPPC membrane. This is in good agreement with the DSC measurements, in which no pretransition was observed for all mixed systems, showing that no ripple phase is formed. Increasing the content of unsaturated lipid to *X*_DOPC_ = 0.3 causes its molecules to cluster together to form domains. Their thickness is much smaller compared to the membrane fragments composed mainly of DPPC. This indicates that a mixed membrane of this composition is a biphasic system, which is consistent with our DSC measurements. The introduction of DOPC into the DPPC membrane results in a significant broadening of the main transition peak, indicating a decrease in the size of cooperative units. DPPC-rich membrane fragments show high ordering, as demonstrated by the *S*_CH_ values; thus, they are in the gel state.

In the case of the bilayer with *X*_DOPC_ = 0.5, our simulations show a clear phase separation that leads to DPPC-rich domains suspended in a DOPC-rich phase. Interestingly, these domains are in register, which means that the domains in one leaflet are coupled to the same domains in the other leaflet. The image of the DPPC/DOPC membrane with *X*_DOPC_ = 0.5 obtained in our simulations (Figure 3) is strongly supported by experimental results. X-ray diffraction measurements of an equimolar mixture of DOPC and DPPC at 30 °C showed that the two phospholipids are completely separated within the bilayer [44]. In addition, the bilayers are completely in register, so that DPPC domains in one leaflet are invariably aligned with DPPC in the opposing leaflet and likewise with DOPC. The DPPC domains are in the gel phase, while the DOPC domains are characterized by the presence of disordered chains. The formation of domains in the DOPC/DPPC (1:1) membrane at room temperature has also been demonstrated directly by AFM visualizations [47,48]. AFM measures the lateral organization of lipid bilayers deposited on a substrate by imaging the topographical details of the membrane in the form of domain lateral size and height relative to surrounding lipids in the liquid state. However, AFM images showed that the substrate affects the domain organization. Micrometer-sized domains with rounded boundaries were formed in the DOPC/DPPC (1:1) bilayers on mica [47]. In contrast, domains with irregular shape and sizes between 100 and 200 nm were observed for the DOPC/DPPC (1:1) bilayer prepared on glass [48]. 

In the DPPC/DOPC membranes with a predominance of DOPCs (*X*_DOPC_ ≥ 7), the phase separation gradually disappears. For the system with 70 mol% of the unsaturated lipid, we observed the formation of small aggregates, which is strongly supported by the DSC measurements. However, the properties of both lipids such as surface area per lipid (APP and APO) are comparable. This indicates that the boundary between the system being monophasic or biphasic is quite blurred. This can be the reason for the discrepancies in the phase diagrams determined by different experimental methods. 

We also focused on the arrangement of the headgroups of both lipids in the mixed membranes. The results show that the phosphocholine groups have a lot of freedom in orientation but mainly make an angle in the range of 60–90° to the bilayer normal, which means that they are aligned almost parallel to the membrane surface. This orientation of the headgroups is almost independent of temperature and membrane composition. This agrees with neutron-scattering experiments, which report that the average orientation of the phosphocholine group of DPPC in the gel state, as well as in the liquid-crystalline state, is almost parallel to the membrane surface [49]. Semchyschyn and Macdonald reported that for 1,2-dimyristoyl-*sn*-glycero-3-phosphocholine (DMPC), the headgroup vector is about 0.45 nm in length and oriented at an angle of about 30° above the bilayer surface [50]. The parallel alignment of the PC headgroup to the membrane surface can be achieved by two different conformations within the glycerol group. The *gauche* configuration of the CG-CG-CG-O dihedral angle forces the phosphocholine group to point away from the rest of the molecule (conformer A), while in the *trans* configurations the headgroup is located above the lipid molecule (conformer B). Our simulations indicate that in the gel phase, the contribution of conformer B is about 70% and decreases slightly when the membrane is in the liquid phase. Our simulation results are in line with experimental findings. Using two-dimensional ^13^C-^1^H chemical shift correlation spectroscopy, it was shown that the arrangement of the DMPC molecule, in which its headgroup and the beginning of the *sn*-2 chain bend toward each other, is significantly populated in the liquid bilayer, suggesting that conformer B dominates in the liquid-crystalline phase [51].

**The driving forces for phase separation in the DPPC/DOPC systems**. The driving forces for phase separation between different lipids in the bilayer have long been identified as resulting from adverse interactions between lipid molecules. It is assumed that the miscibility of lipids depends mainly on the difference in the interaction energies among the like-pairs and mixed-pairs formed in the mixed membrane [12,28]. Lateral phase separation and domain formation can occur when like-pair interactions are stronger than those of the mixed-pairs. Interactions between lipid molecules occur both in their hydrophobic part and between their headgroups. The hydrophobic part consists of hydrocarbon chains, which can vary in length and degree of unsaturation. The forces acting in the hydrocarbon region of the bilayer are dominated by van der Waals attractions between the acyl chains. In contrast, lipid headgroups interact with each other through electrostatic and van der Waals interactions. 

Our experimental results show that the free excess energy of mixing of DPPC and DOPC is positive at 22 °C, indicating that the miscibility of these lipids is thermodynamically unfavorable. The Gibbs–Helmholtz equation for the free excess Gibbs energy of mixing is giving by the following equation:

ΔG^Exc^ = ΔH^Exc^ − *T* ΔS^Exc^ (1)

where ΔH^Exc^, ΔS^Exc^, and *T* are the enthalpy and entropy of mixing and the temperature, respectively. Thus, the free excess energy of mixing is divided into an entropic component, mainly due to the loss of order of the hydrocarbon chains, and the enthalpic component, resulting mainly from interactions between lipid molecules. Our simulations showed that the electrostatic and dispersive components of the interaction energy in the DPPC/DOPC bilayer are almost constant with temperature change. In addition, the interaction energies for lipid like-pairs are more than 75 times stronger than for mixed-pairs. Therefore, we believe that the change in miscibility of these two lipids with increasing temperature is related to the entropic component. The entropy gain arises due to an increase in the disorder of the acyl chains above the transition temperature *T*_m_. As a result, the value of the *T* ΔS^Exc^ term increases with increasing temperature, causing a decrease in ΔG^Exc^_,_ and making the miscibility in the DPPC/DOPC system thermodynamically favorable. 

## 4. Conclusions

In this work, we investigated the molecular organization of lipid bilayers composed of PC with oleoyl and palmitoyl chains. The experimental results showed that DOPC/DPPC mixed systems exhibit very limited miscibility (strongly positive values of the excess free energy of mixing) at temperatures below the DPPC phase transition. After melting the saturated lipid, the DPPC/DOPC membranes are single-phase systems. This indicates that the presence of DPPC above 10 mol% in the membrane (e.g., DPPC is the main phospholipid in pulmonary surfactant composition) will result in the spontaneous formation of domains of this lipid at physiological temperatures. The MD simulations show that although the two lipids share the same headgroup, electrostatic interactions for lipid like-pairs are much stronger than for mixed-pairs, and temperature has only a small effect on these interactions. The reason for this may be the different depth of localization of the polar groups of the two lipids; the DOPC headgroups are located much deeper in the mixed bilayer compared to the saturated lipid, both in the gel and liquid states. Therefore, the explanation for the change in solubility in the saturated–unsaturated lipid system with increasing temperature should be traced to the entropic component of the excess free energy of mixing. As a result of the melting of the palmitoyl chains of DPPC, the disordering of the hydrophobic region of the membrane increases significantly, and therefore the entropy of the system increases. Consequently, the entropic component increases strongly with increasing temperature, resulting in a decrease in the value of the excess free energy of mixing. Thus, the miscibility of phospholipids with different saturations of acyl chains is an entropy-driven process.

## Figures and Tables

**Table 1:** Detailed description of the compositions of the simulated systems.

System	DPPC	DOPC	Water	# of Repeats × Simulation Length
DPPC	128	0	6400	3 × 1 µs
DPPC/DOPC, *X*_DOPC_ = 0.1	114	14	6400	3 × 1 µs
DPPC/DOPC, *X*_DOPC_ = 0.3	90	38	6400	3 × 1 µs
DPPC/DOPC, *X*_DOPC_ = 0.5	64	64	6400	3 × 1 µs
DPPC/DOPC, *X*_DOPC_ = 0.7	38	90	6400	3 × 1 µs
DPPC/DOPC, *X*_DOPC_ = 0.9	14	114	6400	3 × 1 µs
DOPC	0	128	6400	3 × 1 µs

**Table 2:** Calculated average values of the area per lipid (APL) in the lipid membranes at 295 K and 323 K. Errors, estimated as the standard deviation of the averages, are smaller than 0.02 nm^2^. Calculated averaged values of surface area per DPPC (APP) and area per DOPC (APC) in the lipid membranes at 295 K and 323 K. Errors, estimated as the standard deviation of the averages, are smaller than 0.045 nm^2^.

System	APL (nm^2^)		APP (nm^2^)		APO (nm^2^)	
	295 K	323 K	295 K	323 K	295 K	323 K
DPPC	0.567	0.664	0.569	0.665	-	-
DPPC/DOPC, *X*_DOPC_ = 0.1	0.576	0.677	0.572	0.676	0.608	0.676
DPPC/DOPC, *X*_DOPC_ = 0.3	0.594	0.693	0.586	0.693	0.603	0.694
DPPC/DOPC, *X*_DOPC_ = 0.5	0.668	0.710	0.664	0.707	0.670	0.710
DPPC/DOPC, *X*_DOPC_ = 0.7	0.700	0.725	0.701	0.723	0.700	0.726
DPPC/DOPC, *X*_DOPC_ = 0.9	0.715	0.738	0.712	0.736	0.714	0.737
DOPC	0.722	0.744	-	-	0.723	0.744

## Data Availability

The data presented in this study are available on request from the corresponding authors.

## Supplementary Materials

The following supporting information can be downloaded at: https://www.mdpi.com/article/10.3390/membranes13040411/s1. Figure S1: DSC heating thermograms for the DPPC/DOPC aqueous dispersions as a function of the DOPC mole fraction. Figure S2: Time evolution of thermodynamic and structural parameters from a 1-µs long MD simulation of system DPPC/DOPC with *X*_DOPC_ = 0.9 at 295 K. Representative plots of total energy (A), system volume (B), system density (C), and area per lipid (D) depicting the convergence of the simulations are shown. Area per lipid was calculated as the area of the simulation box divided by the number of lipids in one leaflet. The running average of the data is shown as a dark green line, while the original data are presented in light green. Figure S3: Representative snapshots from the MD simulations showing the organization of lipid membranes with different lipid compositions at 323 K from the side (left) and top (right). Lipids are shown in the licorice representation. DOPC lipids are shown in orange (hydrocarbon chains) and red (headgroups), while DPPC lipids are shown in blue (hydrocarbon chains) and yellow (headgroups). Water is not shown for clarity. Figure S4: Probability distributions of the tilt angles θc between the sn-1 lipid chain vector (shown in Figure 1) and the bilayer normal for DOPC (A) and DPPC (B) and probability distributions of the angle *θ*_h_ between the headgroup vector and the bilayer normal for DOPC (C) and DPPC (D) at 310 K. The probabilities were averaged over the last 500 ns of the trajectories. Figure S5: Mass density profiles of selected chemical groups in DPPC/DOPC bilayers at 323 K. The density profiles of water (cyan curve), phosphorus atoms (green and black curves, for DOPC and DPPC, respectively) and the terminal methyl group of hydrocarbon chains (red and yellow curves, for DOPC and DPPC, respectively) are shown. Figure S6: Averaged position of the terminal methyl group of the lipid sn-1 and sn-2 chain along the bilayer normal at 295 K. Figure S7: Averaged position of the terminal methyl group of the lipid sn-1 and sn-2 chain along the bilayer normal at 323 K. Table S1: The thickness (*d*_P_) of bilayers calculated as an average distance between of the phosphorus atoms of the opposite leaflets for each type of lipid at 295 K and 323 K. Errors are estimated as the standard deviation of the averages.
