# Supplementary material for: Miscibility of Phosphatidylcholines in Bilayers: Effect of Acyl Chain Unsaturation
Source: Membranes (Basel). 2023 Apr 5;13(4):411. doi: 10.3390/membranes13040411 (PMC10146409; doi:10.3390/membranes13040411)
Supplement: Supplementary file 1 [file membranes-13-00411-s001.zip › membranes-2283301-supplementary.pdf]

Supplementary Materials for

Miscibility of Phosphatidylcholines in Bilayers: Effect of Acyl Chain Unsaturation

Agata Żak [1], Natan Rajtar [1], Waldemar Kulig [2,*] and Mariusz Kepczynski [1,*]

1 Faculty of Chemistry, Jagiellonian University, Gronostajowa 2, 30-387 Kraków, Poland
2 Department of Physics, University of Helsinki, P.O. Box 64, FI-00014 Helsinki, Finland
* Correspondence: waldemar.kulig@helsinki.fi (W.K.); kepczyns@chemia.uj.edu.pl (M.K.)

DSC measurements

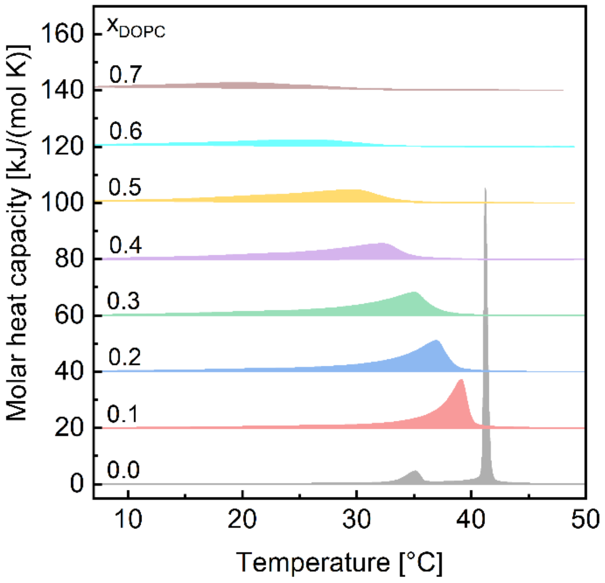

Figure S1. DSC heating thermograms for the DPPC/DOPC aqueous dispersions as a function of the DOPC mole fraction.

MD Simulations

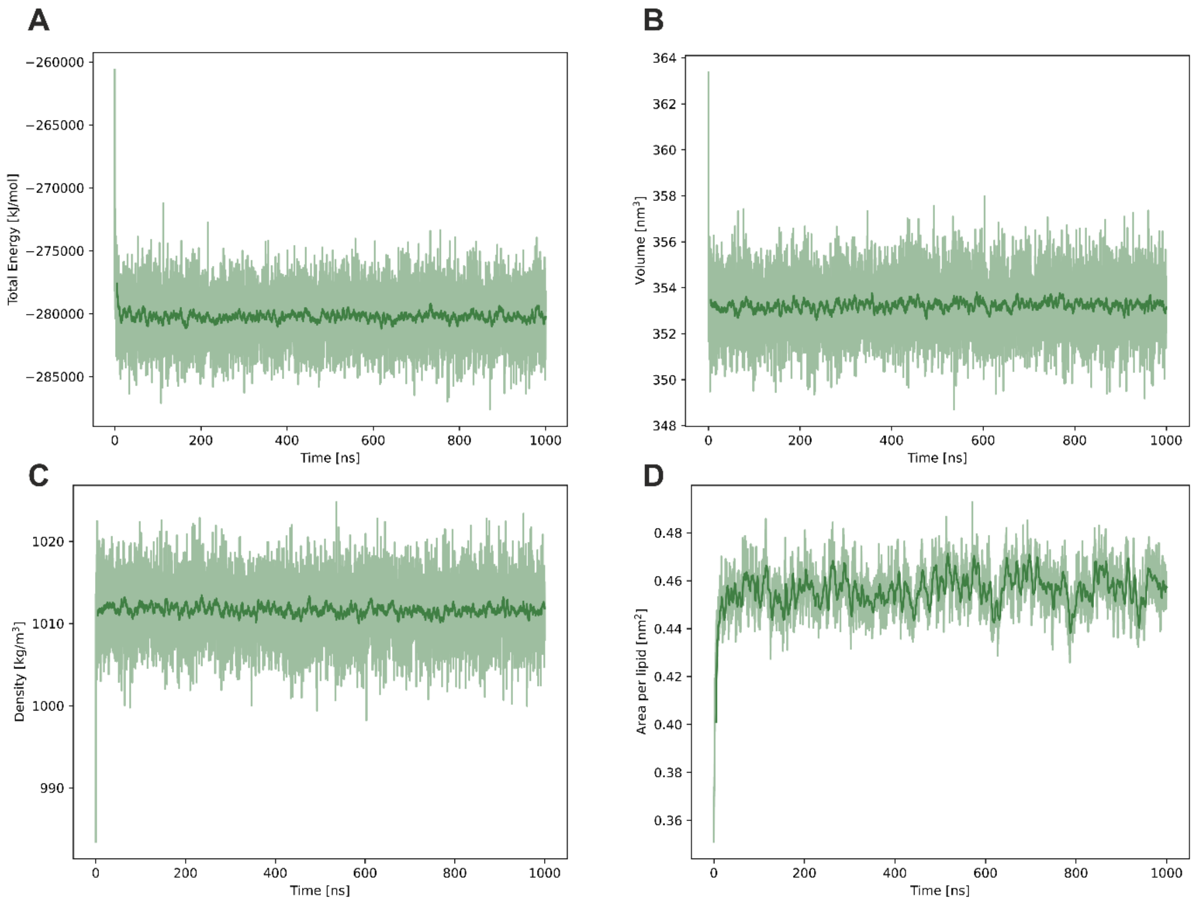

Figure S2. Time evolution of thermodynamic and structural parameters from a 1-μs long MD simulation of system DPPC/DOPC with X_{DOPC} = 0.9 at 295 K. Representative plots of total energy (A), system volume (B), system density (C), and area per lipid (D) depicting the convergence of the simulations are shown. Area per lipid was calculated as the area of the simulation box divided by the number of lipids in one leaflet. The running average of the data is shown as a dark green line, while the original data are presented in light green.

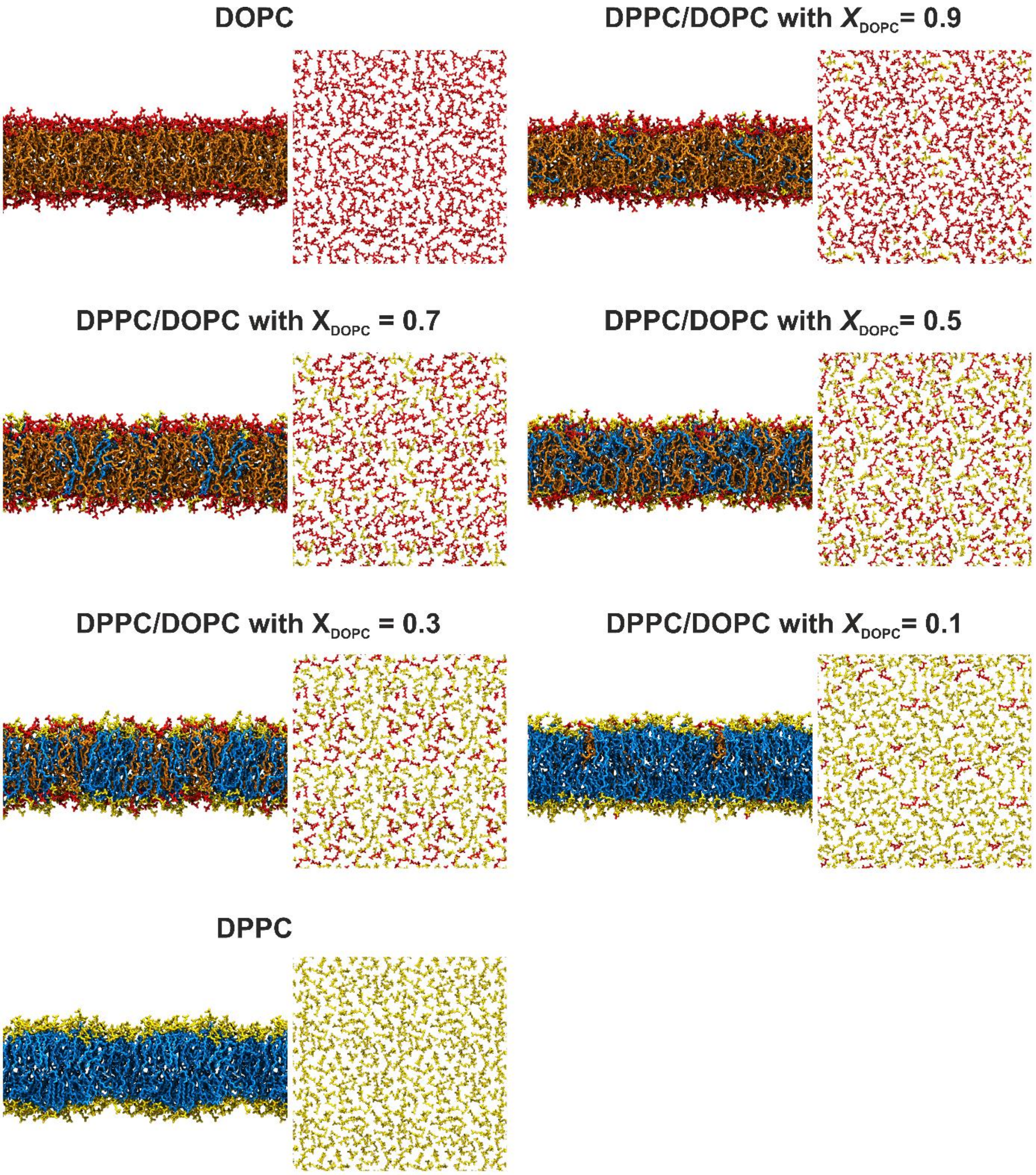

Figure S3. Representative snapshots from the MD simulations showing the organization of lipid membranes with different lipid compositions at 323 K from the side (left) and top (right). Lipids are shown in the licorice representation. DOPC lipids are shown in orange (hydrocarbon chains) and red (headgroups), while DPPC lipids are shown in blue (hydrocarbon chains) and yellow (headgroups). Water is not shown for clarity.

Table S1. The thickness (d_P) of bilayers calculated as an average distance between of the phosphorus atoms of the opposite leaflets for each type of lipid at 295 K and 323 K. Errors are estimated as the standard deviation of the averages.

System	d_P for DPPC [nm]		d_P for DOPC [nm]	
	295 K	323 K	295 K	323 K
DPPC	4.02 ± 0.09	3.72 ± 0.01		
DPPC/DOPC, X_{DOPC} = 0.1	4.13 ± 0.05	3.71 ± 0.01	3.60 ± 0.06	3.80 ± 0.02
DPPC/DOPC, X_{DOPC} = 0.3	4.30 ± 0.07	3.71 ± 0.00	3.60 ± 0.04	3.69 ± 0.01
DPPC/DOPC, X_{DOPC} = 0.5	3.92 ± 0.10	3.74 ± 0.02	3.67 ± 0.01	3.65 ± 0.00
DPPC/DOPC, X_{DOPC} = 0.7	3.80 ± 0.02	3.77 ± 0.01	3.66 ± 0.01	3.64 ± 0.00
DPPC/DOPC, X_{DOPC} = 0.9	3.88 ± 0.03	3.93 ± 0.07	3.66 ± 0.01	3.64 ± 0.00
DOPC			3.66 ± 0.00	3.64 ± 0.00

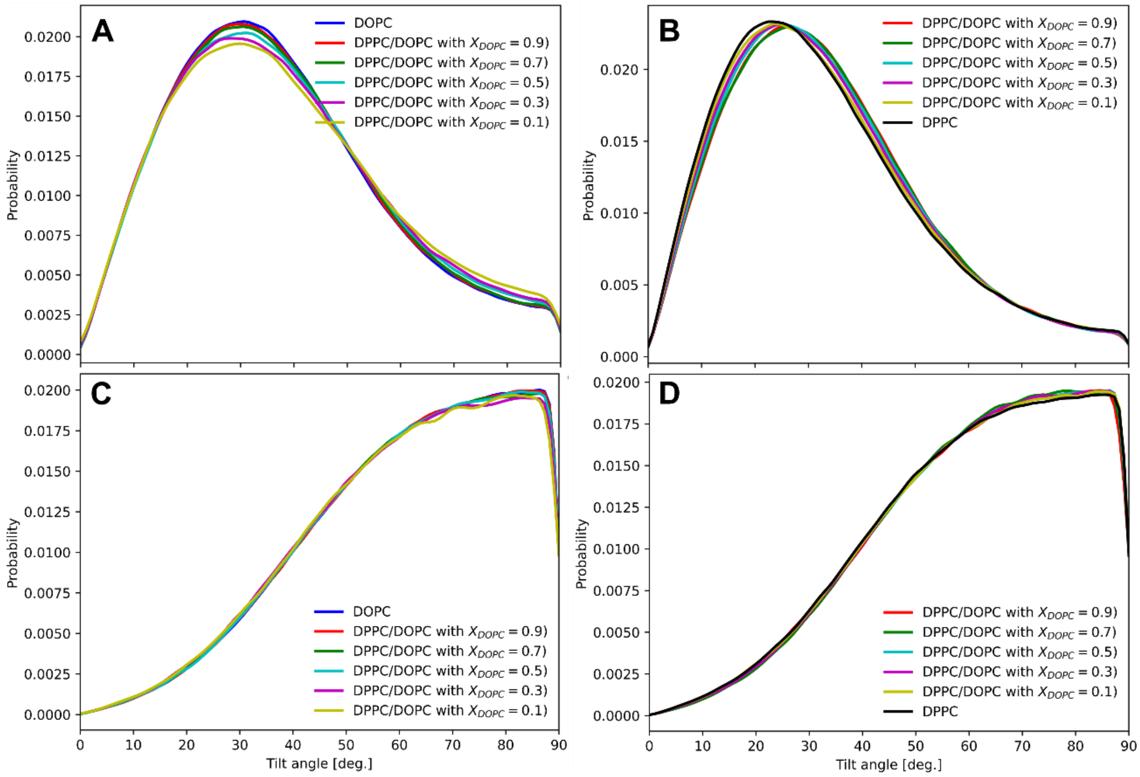

Figure S4. Probability distributions of the tilt angles θ_c between the *sn*-1 lipid chain vector (shown in Figure 1) and the bilayer normal for DOPC (A) and DPPC (B) and probability distributions of the angle θ_h between the headgroup vector and the bilayer normal for DOPC (C) and DPPC (D) at 310 K. The probabilities were averaged over the last 500 ns of the trajectories.

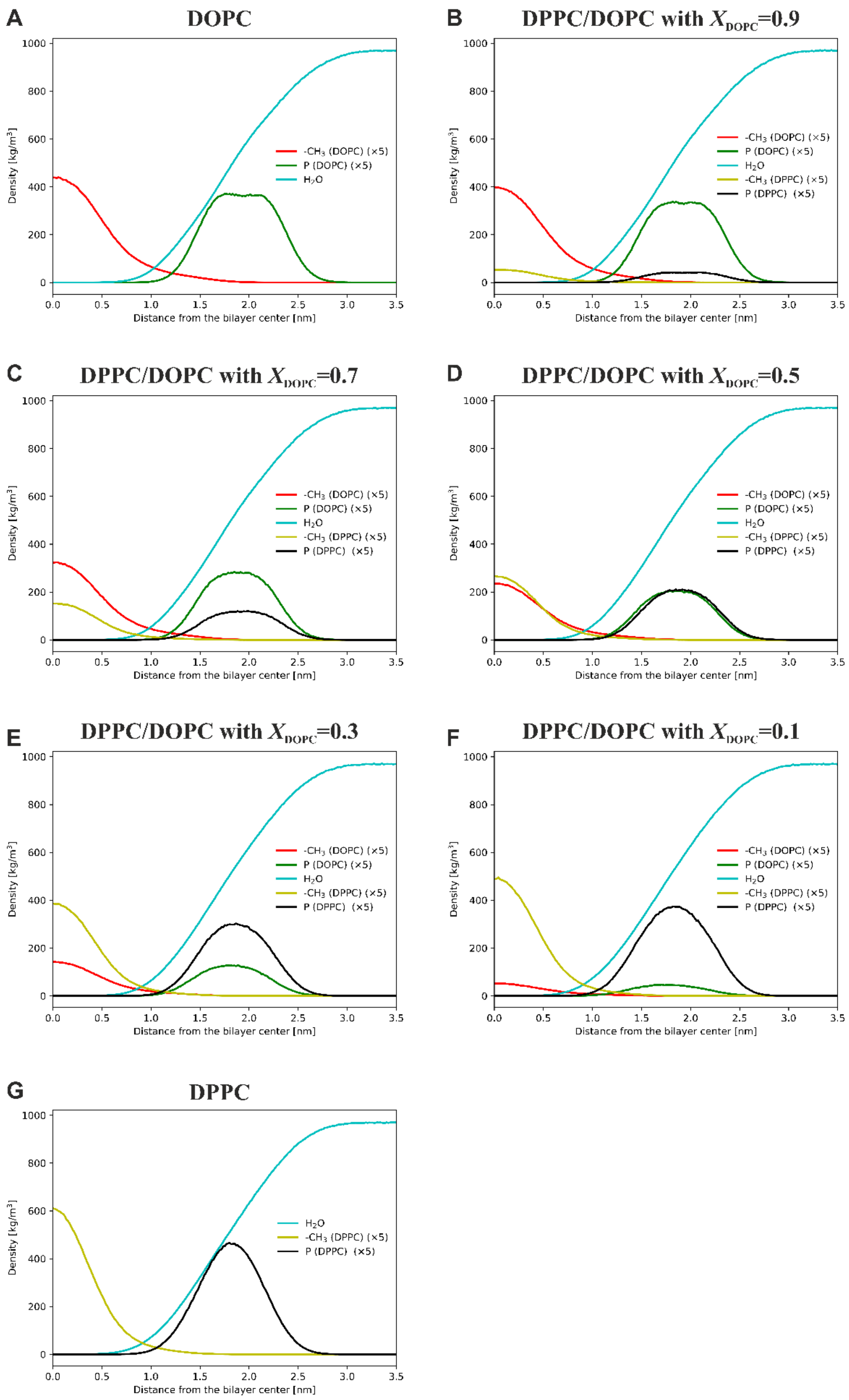

Figure S5. Mass density profiles of selected chemical groups in DPPC/DOPC bilayers at 323 K. The density profiles of water (cyan curve), phosphorus atoms (green and black curves, for DOPC and DPPC, respectively) and the terminal methyl group of hydrocarbon chains (red and yellow curves, for DOPC and DPPC, respectively) are shown.

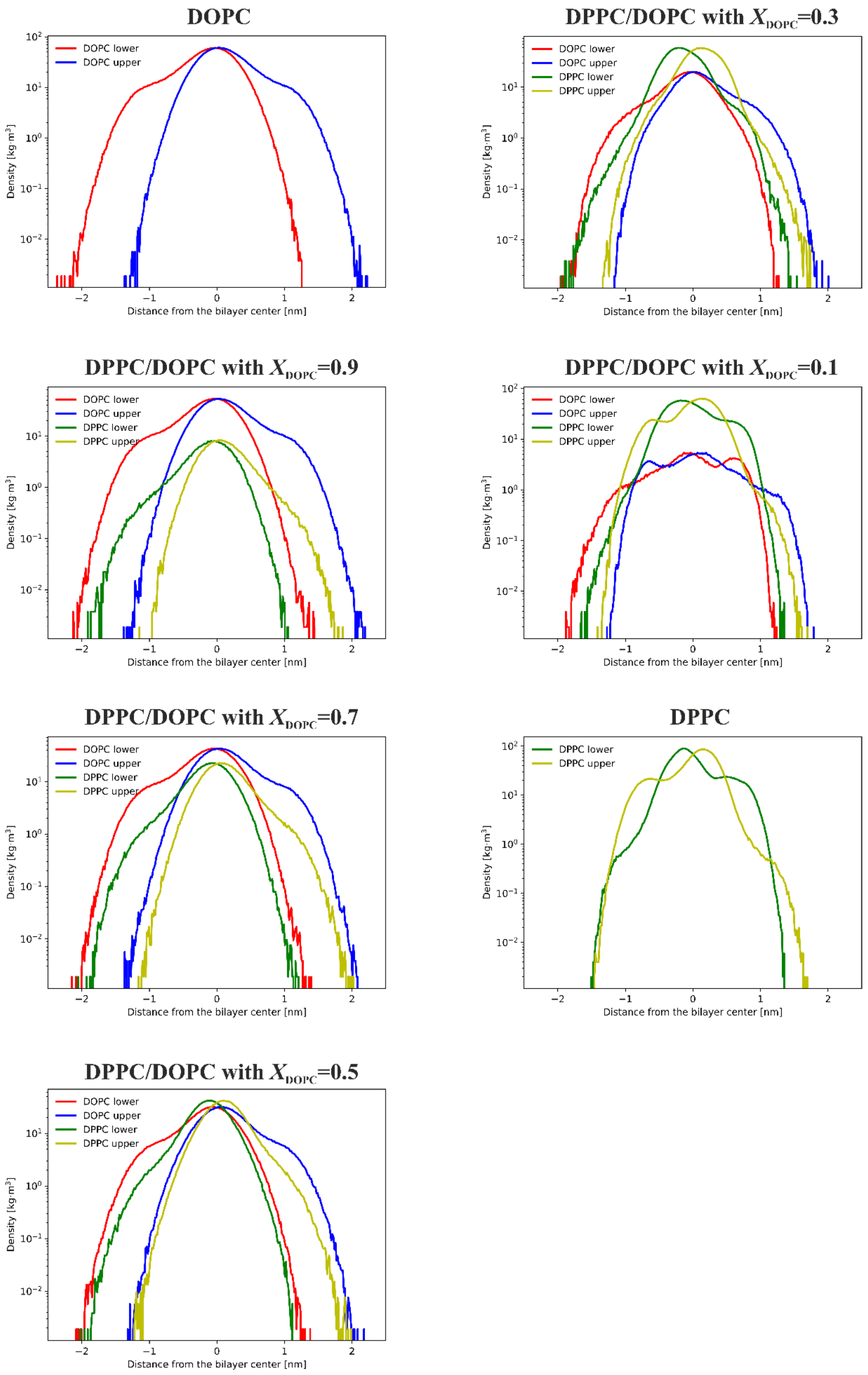

Figure S6. Averaged position of the terminal methyl group of the lipid *sn*-1 and *sn*-2 chain along the bilayer normal at 295 K.

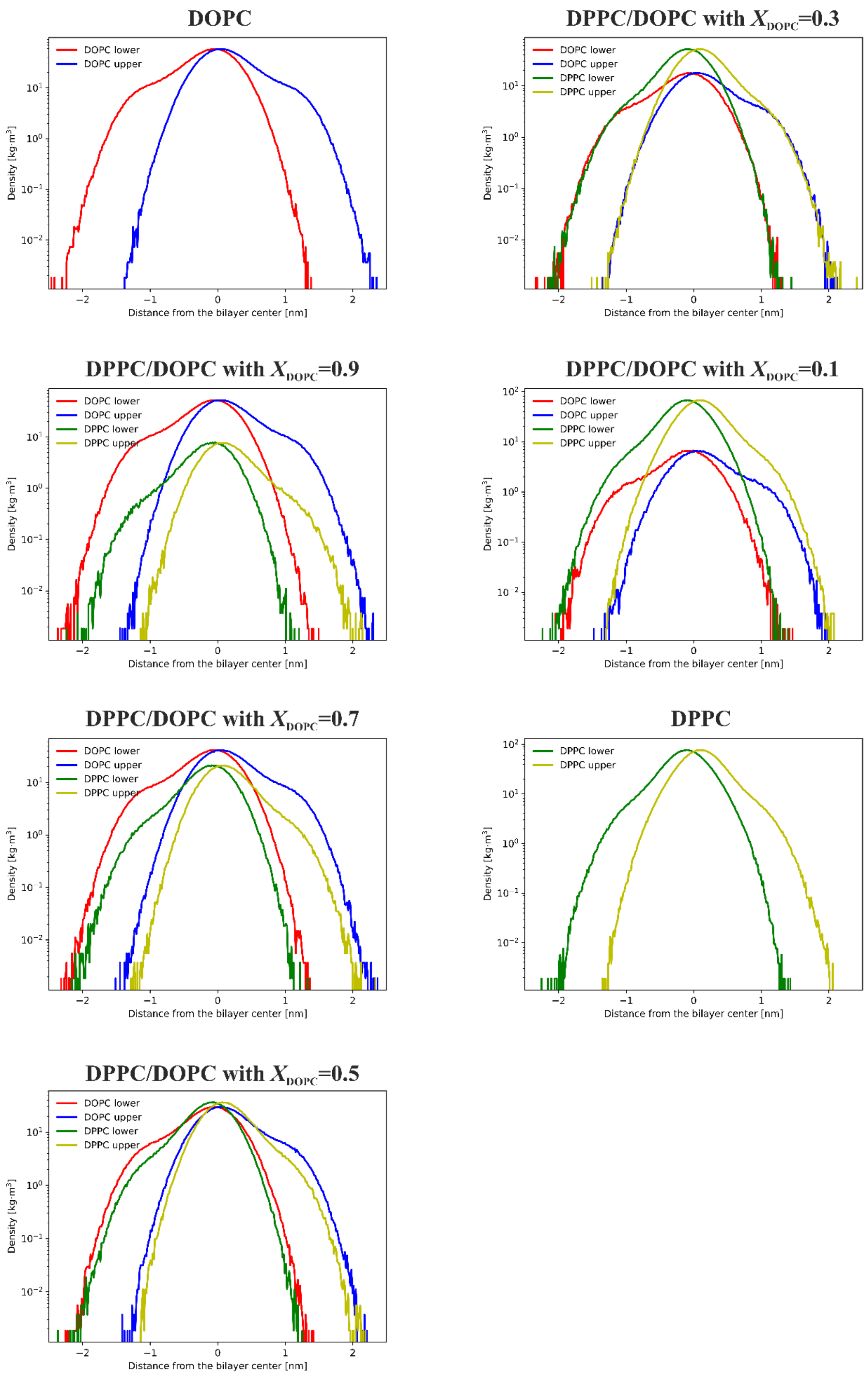

Figure S7. Averaged position of the terminal methyl group of the lipid *sn*-1 and *sn*-2 chain along the bilayer normal at 323 K.

ACKNOWLEDGEMENTS

The project was financed by the National Science Centre. Poland (grant no. 2019/35/B/ST5/02147). The authors would also like to acknowledge the computing resources provided by the CSC – IT Center for Science Ltd. (Espoo, Finland).
